# Comparison of alternate references for femoral rotation in female patients undergoing total knee arthroplasty

**DOI:** 10.1007/s00167-015-3506-y

**Published:** 2015-01-20

**Authors:** Hyung-Min Ji, Dong San Jin, Jun Han, Ho-Sik Choo, Ye-Yeon Won

**Affiliations:** Department of Orthopaedic Surgery, Ajou University of College of Medicine, 164, World Cup-ro, Yeongtong-gu, Suwon, 443-721 South Korea

**Keywords:** Knee replacement arthroplasty, Humans, Female, Knee joint, Femur, X-ray computed tomography, Three-dimensional imaging

## Abstract

**Purpose:**

Accurate rotational alignment of the femoral component is of vital importance for successful total knee arthroplasty (TKA). Two anatomical references located on the anterior femur were recently introduced. To determine which is more reliable reference axis for the femoral component rotation in female patients receiving TKA, the trochlear anterior line was compared with the femoral anterior tangent line.

**Materials and methods:**

Preoperative computed tomography in 76 patients receiving TKA for varus deformity was performed, and the images were reconstructed into three-dimensional models. The trochlear anterior line was defined as the line connecting the most anterior portion of the lateral and medial femoral condyles and the femoral anterior tangent line as the line parallel to distal anterior femoral surface. The two angles between these reference axes and the surgical transepicondylar axis (TEA) in three-dimensional images (trochlear anterior line/TEA, femoral anterior tangent line/TEA) were measured. The correlation between these two angles was computed. We investigated to see whether a significant difference in variance existed.

**Results:**

The trochlear anterior line was internally rotated by 6.1° ± 2.5° with respect to TEA, whereas the femoral anterior tangent line by 9.5° ± 3.8°. The trochlear anterior line was externally rotated by 3.4° ± 3.3° with respect to the femoral anterior tangent line. There was a significant correlation between the trochlear anterior line/TEA and the femoral anterior tangent line/TEA.

**Conclusions:**

The variance of the trochlear anterior line/TEA was significantly smaller than that of the femoral anterior tangent line/TEA demonstrating a more consistent distribution. When conventional reference axes such as the posterior condylar axis or the anteroposterior axis are unclear or differ, surgeons can rely on these alternative references. When trochlear anterior line and femoral anterior tangent line contradicts, the former might be more reliable for the rotational alignment of the femoral component in female patients.

**Level of evidence:**

Case series with no comparison group, Level IV.

## Introduction

Appropriate rotational alignment of the femoral component is essential for successful total knee arthroplasty (TKA) as well as long-term survival of the implant itself [1, 3]. This is because the rotational alignment of the femoral component not only affects tracking of the patellar component but also determines the flexion gap of the femoral component [2, 13]. Previous studies suggested using the posterior condylar axis (PCA) [12], Whiteside’s line [18] or the transepicondylar axis (TEA) [9, 11, 14, 20] as a reference axis for determining rotational alignment of the femoral component, and there have been studies on the angles created between these reference axes [8, 12, 15, 16, 18]. However, it is not always easy to apply such traditional references in the operative field because arthritic changes such as deformities, bony defects and osteophytes not only make it difficult to identify these references but also distort them [3, 5].

Recently, researchers proposed two reference axes in the anterior femur as alternatives when conventional reference axes are ill-defined or distorted. The trochlear anterior line (TAL) is the line which connects the anterior points of greatest protrusion of the femoral medial and lateral condyles [6, 8, 19], whereas the femoral anterior tangent line (FAT) is a line parallel to the anterior surface just proximal to the point where the femoral trochlea ends [15, 16]. Both reference axes can be used to determine the rotational alignment of the femoral component and have been regarded as useful indices [8, 15–17, 19]. They are located in the anterior aspect of the femur and anatomically close hence determining the relative spatial relationship is relatively facilitating. Studies regarding the relative position of the two reference axes not only provide valuable supplemental information for determining rotational alignment of the femoral component but also can serve as key anthropometric data of the anterior distal femur and provide useful information when designing implants. Despite the potential significance, there have been virtually no studies to date comparing these two reference axes. The purpose of this study was to determine the relative spatial correlation between the TAL and the FAT and to elucidate which reference axis might be more reliable by comparing variances between the two lines.

## Materials and methods

Seventy-six consecutive Korean patients that received TKA from October 2011 to April 2012 for osteoarthritis of the knee at our institution were selected. No patient was excluded because of age and gender. Patients were excluded if they had had a previous bony surgery or replacement that might have changed femoral geometry. The average age was 70.3 ± 6.0 years (range 50–85). The average preoperative mechanical axis deviation (MAD) was 10.5° ± 5.3°. These were all women.

**Fig. 1:**
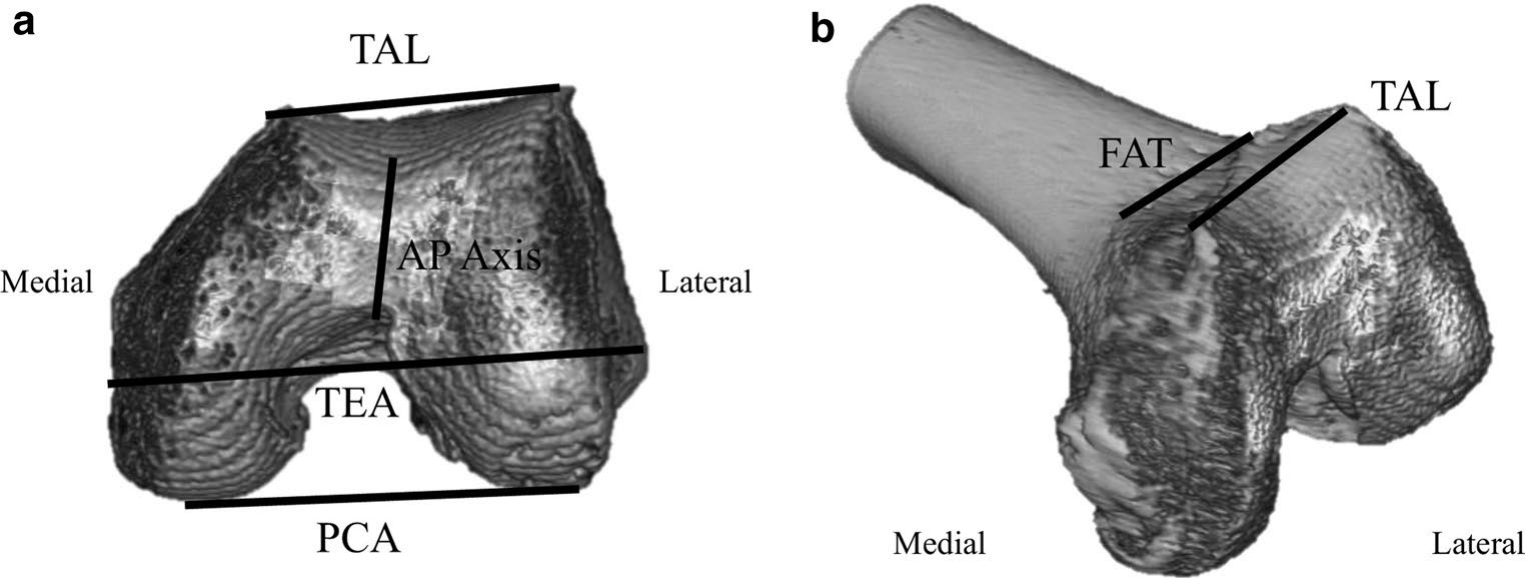
Traditional and additional alternative references for femoral component rotation are depicted. **a** 3D-reconstructed distal femur seen from below. **b** Distal femur seen from the front in an oblique direction. *TEA* transepicondylar axis, *AP* anteroposterior, *PCA* posterior condylar axis, *TAL* anterior trochlear line and *FAT* femoral anterior tangent line

On both knees prior to the operation, 2-mm sliced computed tomography (CT) (Siemens Ltd., Erlangen, Germany) was performed. The images were scanned centring the knee joint using a 512 × 512 pixel matrix at a thickness of 2 mm for a length of 200 mm, obtaining more than 100 sequential images in total, and these were exported to a software program (Xelis software, version 1.0.2.2; Infinitt, Seoul, Korea) to create three-dimensional images. The tibia, patella, as well as osteophytes from the images were omitted to facilitate simulation and observe anatomical indices. This computer software allowed us to create a three-dimensional model from two-dimensional images and depict lines and dots on specific areas of the model, which could be transposed back onto the two-dimensional images. Angles between two lines could also be measured. As described in previous methods, the TAL was defined as a line connecting the anterior aspects of greatest protrusion of the femoral medial and lateral condyles (Fig. 1a) [8, 19]. The FAT was defined as a line parallel to the anterior surface of the distal femur in the axial plane where the femoral trochlea begins (Fig. 1b) [15, 16]. Based on previous studies, the surgical TEA was defined as the line connecting the most prominent lateral epicondylar projection and medial epicondylar groove, the AP axis as the line connecting the deepest point of the patellar groove and the point of the intercondylar notch, and the PCA as the line connecting points between the articular cartilages of both femoral posterior condyles [14, 18, 20]. All of these lines could be superimposed in any axial plane, and the angle between these axes could be measured by using the functions embedded in the software. The angle between the TAL and the TEA was defined as TAL/TEA and that between the FAT and TEA as FAT/TEA. We also measured the angle between the TAL and FAT (TAL/FAT), PCA and TEA (PCA/TEA), and Whiteside’s line and TEA (AP/TEA). Two independent observers (HMJ and DSJ) measured all angles, and one observer (HMJ) evaluated 4 weeks apart to assess the inter-observer and intra-observer reproducibility. The inter-observer reproducibility was 0.832, 0.875, 0.845, 0.864 and 0.858, respectively for TAL/TEA, FAT/TEA, TAL/FAT, PCA/TEA and AP/TEA. The intra-observer variability was 0.902, 0.921, 0.893, 0.917 and 0.897, respectively. We tried to decide whether any correlation existed between the TAL/FAT and the preoperative MAD and the age of the patients prior to the operation. The correlation between the TAL/TEA and the FAT/TEA was calculated, and their variance was compared to determine which angle had smaller variance.

This retrospective study was approved by the institutional review board of our hospital (IRB approval, Ajou University Hospital, MED-MDB-14-173).

### Statistical analysis

All numbers were calculated to the second decimal place and presented to the first after raising the second. A sample size of 75 patients with CT scan would provide sufficient power (>80 %) to show differences of variances between the TAL and FAT >10 % as statistically significant (two-tailed *α* = 0.05). All demographic data and measured angles were shown to fall into a normal distribution, and all statistical values were illustrated as average and standard deviation. Correlation analysis performed using Pearson’s correlation coefficient, which is in general subcategorized as poor (0.00–0.20), fair (0.21–0.40), moderate (0.41–0.60), good (61–0.80) and perfect (0.81–1.00) [7]. The Pearson’s correlation coefficient was also used to evaluate the inter-observer and intra-observer reproducibility of all measurements. The variances between the angles TAL/TEA and FAT/TEA were compared by using *F*-test. A *p* value <0.05 was defined as statistically significant. All statistical analysis was performed using SPSS Ver. 14.0 (SPSS Inc., Chicago, USA).

## Results

**Fig. 2:**
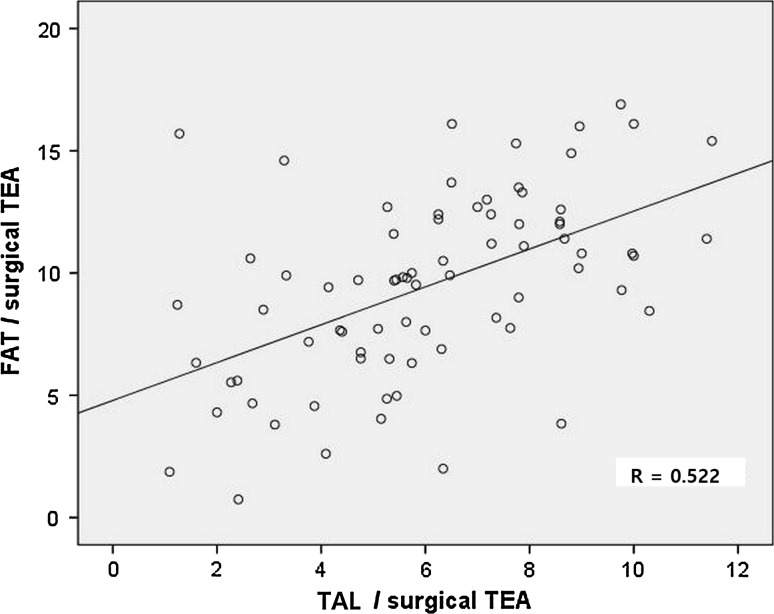
Relationships between TAL/TEA and FAT/TEA. The angle between the TAL and surgical TEA is observed to correlate with the TAL and FAT (*r* = 0.52; *p* < 0.001)

**Fig. 3:**
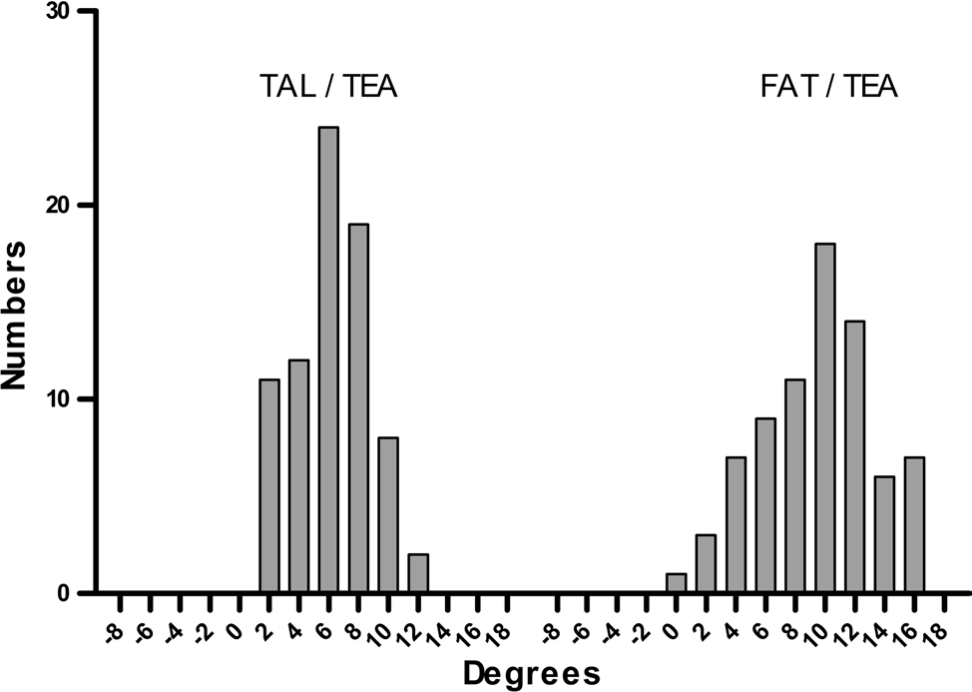
Distribution of the angles between the TAL and surgical TEA (TAL/TEA) and between the FAT and surgical TEA (FAT/TEA). The variability of the FAT line and TAL line to the clinical TEA was different (*α* = 0.002)

The TAL was internally rotated with respect to the TEA by 6.1° ± 2.5°, whereas the FAT by 9.5° ± 3.8°. The FAT was internally rotated by 3.4° ± 3.3° with respect to the TAL. The PCA was internally rotated by 2.7° ± 1.2° with respect to the TEA, and the line perpendicular to the AP axis was externally rotated by 1.3 ± 3.8 with respect to the TEA. There was no significant correlation between FAT/TAL and the MAD and age [MAD: *r* = 0.053 (n.s.), age: *r* = 0.136 (n.s.)]. Correlation between TAL/TEA and FAT/TEA was moderate (*r* = 0.520; *p* < 0.001) (Fig. 2). We compared the distribution of the two angles using histograms (Fig. 3). When comparing variances between the TAL/TEA and FAT/TEA, the *α* value was 0.002, demonstrating that the variance of the TAL/TEA was significantly smaller than that of the FAT/TEA and hence signifying that the TAL/TEA had a relatively more homogenous distribution.

## Discussion

The most important finding of the present study was that TAL has a relatively more homogenous distribution than FAT based on comparison of two novel anatomical references located on anterior femoral cortex for femoral rotation during TKA in female patients. FAT is internally rotated by 3.4° ± 3.3° with respect to the TAL. This study is to our knowledge unprecedented in reporting the relative spatial correlation between the FAT and the TAL. A number of authors agreed that the TEA is the anatomical reference axis for the rotational alignment of the femoral component [2, 3, 5, 12], and biomechanical studies have elucidated that the surgical TEA falls on the centre of knee rotation [4]. The FAT, as in our study, has been reported to be internally rotated by 12.2° ± 3.6° with respect to the clinical TEA in previous CT-based studies [16]. In this study, authors did not report the angle between the surgical and clinical TEA. However, the condylar twist angle, which is the angle formed by the PCA and the clinical TEA, was 5.7° ± 2.8° and considering the angle between PCA and surgical TEA to be around 3° the FAT/TEA should be 9.2° ± 3.6°. This is quite close to what we have obtained in this study, which was 9.5° ± 3.8°. The TAL was reported to be internally rotated by 7.3° ± 1.8° with respect to the TEA in healthy knee joints, which is about 1° more internally rotated than what was obtained in our study [19]; however, in patients with arthritis, this has been reported to be 5.6° ± 2.3° [8], which is comparable to our results.

The values of FAT/TAL were fairly homogenously distributed without showing any correlation with either age or the degree of preoperative varus deformity of the knee joint. As confirmed in previous studies, both the FAT and the TAL showed relatively consistent distribution, providing evidence that these are reliable reference axes that could be applied intraoperatively irrespective of preoperative MAD [8, 16].

There was a moderate correlation between the angles of TAL/TEA and FAT/TEA. This finding reveals key points with regard to anatomy of the anterior aspect of the distal femur; although the size of the medial and lateral anterior condyle is increased distally, the degree of internal rotation of the anterior surface of the distal femur with respect to the TEA remains relatively constant. Depending on the rotation of anterior cortical surface where the FAT is measured the degree of protrusion of the anterior forefront of the medial and lateral condyles determined. When comparing variances between the FAT and the TAL, the latter demonstrated a more consistent distribution. The anterior protrusion of the lateral condyle is universally more anterior and bigger than that of the medial condyle, so the distribution of the TAL/TEA is uniform. However, the shape of the FAT tends to be more variable; the shape of the cortical bone is internally rotated where the FAT lies in general with respect to the TEA, but recent cadaveric study showed the median surface of the cortical bone may be depressed or protruded, resulting in negative values of the FAT with respect to the TEA [10]. This may be why the variance of the FAT/TEA tends to be larger than that of the TAL/TEA, implying that the TAL is more reliable as an indicator of rotational alignment. Researchers showed surgeons can use FAT as a reliable alternative reference line with a simple apparatus [17]. This jig also might be used for TAL without any modification.

There are some limitations in this the results to be mentioned. First of all, as all the individuals involved in this study were Korean patients, clinicians should consider ethnic differences. Secondly, as this study was based on 3D graphical representation, we were able to find accurate anatomical indices by using axial planes and three-dimensional images with computer simulation. It is sometimes difficult to find the exact TAL intraoperatively. This is due to the fact that the most anteriorly protruding points may change depending on the surgeon’s level of view and the degree of knee joint flexion. As all previous studies dealing with TAL, as well as ours, were based on CT images [16, 19], it should be validated whether the TAL can be measured in a reproducible manner intraoperatively in future studies. Thirdly, current study carried limitations as only elderly female patients with varus deformity were included causing restrictions when applying our results to the general population. However, majority of the patients receiving TKA in our institution are old women. One recent study showed medial condyles of Asian females are relatively larger than those of males [20]. Previous study showed FAT was not affected by previous varus–valgus status [16] while TAL was more internally rotated in healthy and valgus knee [8]. These discrepancies should be cleared in the following study.

Despite these limitations, surgeons can rely on these alternative anatomical references when posterior condylar surface and trochlear groove are worn and distorted. Surgeons usually compare the Whiteside line, the PCA and the TEA for more accurate rotational alignment of the femoral component. When one or more of these conventional references are hard to recognize, anterior references such as the FAT or the TAL can be evaluated. As the TAL is located more close to the articular surface, it is more easily accessible than the FAT. This is important when surgeon try to operate minimal invasive TKA. More soft tissue stripping is required to access the FAT, and the result of this study suggests that such additional exposure is not always necessary because the TAL is more uniformly distributed and reliable.

## Conclusions

The FAT was internally rotated by 3.4° ± 3.3° with respect to the TAL, irrespective of the preoperative MAD, and the TAL/TEA values were more homogenously distributed than those of the FAT/TEA in elderly female patients. Therefore, TAL is more reliable as an alternative reference for femoral rotation than FAT. Such information can be useful for determining rotational alignment of the femoral component when conventional reference axes such as the PCA or the AP axis are unclear or largely differ.
